# Complete Genome Sequences of Bordetella pertussis Clinical Isolate FR5810 and Reference Strain Tohama from Combined Oxford Nanopore and Illumina Sequencing

**DOI:** 10.1128/MRA.01207-18

**Published:** 2018-11-15

**Authors:** Valérie Bouchez, Sarah Louise Baines, Sophie Guillot, Sylvain Brisse

**Affiliations:** a Biodiversity and Epidemiology of Bacterial Pathogens Unit, Institut Pasteur, Paris, France; b National Reference Center for Whooping Cough and Other Bordetella Infections, Institut Pasteur, Paris, France; c Department of Microbiology and Immunology, The University of Melbourne at the Peter Doherty Institute for Infection and Immunity, Melbourne, Australia; University of Maryland School of Medicine

## Abstract

Here, we describe the complete genome sequences of two Bordetella pertussis strains, FR5810, a clinical isolate recovered from the respiratory tract of an infant, and Tohama, a key reference strain for the species. Sequences were obtained using a hybrid approach combining Oxford Nanopore Technologies MinION and Illumina NextSeq 500 sequence data.

## ANNOUNCEMENT

Bordetella pertussis is a Gram-negative bacterial pathogen responsible for the vaccine-preventable disease whooping cough. Whole-genome sequencing (WGS) of clinical isolates of B. pertussis is useful to understand transmission at fine typing resolution scales. Circularization of the chromosomal sequence is needed to understand the dynamics of the numerous insertion sequences that may contribute to genetic adaptation of the pathogen populations toward escape of vaccine-induced immunity.

Here, we report the complete genome sequences obtained for clinical isolate FR5810 (1) and reference strain Tohama (2), using a hybrid assembly strategy combining long- and short-sequence reads. FR5810 was isolated in 2014 from the respiratory tract of a 44-day-old baby and was identified using API20E (bioMérieux) on the basis of morphological characteristics and biochemical properties as B. pertussis.

Isolates were first grown on Bordet Gengou Agar (Becton, Dickinson) supplemented with 15% defibrinated horse blood at 36°C for 3 days and then subcultivated for 24 h at 36°C. Bacteria were suspended in physiological water and pelleted for DNA extraction. For Illumina sequencing, bacteria were lysed in phosphate-buffered saline (PBS) 1×, with lysis buffer (Roche Diagnostics) and proteinase K (Roche Diagnostics), by incubating at 65°C for 10 min and then at 95°C for 10 min. Libraries were constructed using the Nextera XT DNA library preparation kit (Illumina), and sequenced on a NextSeq 500 platform (Illumina) using 2 × 150-bp chemistry. For Nanopore sequencing, genomic DNA was prepared by phenol-chloroform extraction using Phase Lock Gel tubes (Qiagen GmbH) and purified by ethanol precipitation. Libraries were prepared using a 1D ligation sequencing kit (SQK-LSK-108, Nanopore), without fragmentation, and sequenced on the MinION using a FLO-MIN-106 flow cell (Nanopore), following the protocol for 1D genomic DNA (gDNA) long reads without BluePippin (Nanopore).

NextSeq 500 sequencing generated 3,691,944 paired reads for FR5810 and 3,037,288 paired reads for Tohama. MinION sequencing generated 237,860 reads (5.2 Gb) for FR5810 and 49,416 reads (1.3 Gb) for Tohama. The average long-read read lengths were 21,785 bp for FR5810 and 26,212 bp for Tohama.

A hybrid Nanopore-Illumina *de novo* assembly was performed using Unicycler v0.4.4 (3) run under a normal assembly mode with default parameters, including a polishing step with Pilon. This produced one circular chromosome of 4,102,412 bp for Tohama (67.7% G+C content). For FR5810, a long-read only assembly was performed with the first 30 fastq files (out of the 59 files produced during the run), corresponding to 124,000 reads, using Unicycler v0.4.4 (3). This produced one circular chromosome of 4,108,173 bp (67.7% G+C content). This sequence was then polished by mapping the Illumina short reads against the chromosome using Snippy v3.2 (https://github.com/tseemann/snippy), correcting variants, and then remapping in an iterative process until no variants were identified. Both chromosomes were annotated using Prokka v1.9 (4).

The obtained sequence of strain Tohama was compared to its initial sequence (NCBI accession number NC_002929) using progressiveMauve snapshot-2015-02-25 (5), which revealed a very good synteny. The clinical isolate FR5810 was characterized as harboring the following vaccine antigen alleles: *ptxP3* and *ptxA1* for the promoter and subunit 1 of pertussis toxin, respectively, alleles *fim2-1* and *fim3-1* for fimbria-encoding genes, and a PRN2-type pertactin. In addition, comparison to the Tohama genome sequence using progressiveMauve snapshot-2015-02-25 (5) revealed genome insertions, deletions, and multiple genomic rearrangements, as previously observed (6, 7).

This work demonstrates, for the first time to our knowledge, the feasibility of achieving high-quality Bordetella pertussis genome sequences from a hybrid approach combining Nanopore and Illumina sequencing.

### Data availability.

The complete genome sequences were deposited in GenBank under the accession numbers CP031787 and CP031788, corresponding to the sample accession numbers SAMN09862822 and SAMN09862835, respectively. Illumina raw reads files were previously published (1) under BioSample accession numbers ERS1869884 and ERS1869859, and Oxford Nanopore Technologies MinION raw reads files were deposited in SRA under the accession numbers SRX4744160 and SRX4744159. The versions described in this paper are the first versions.
